# Flow dynamic assessment of native mitral valve, mitral valve repair and mitral valve replacement using vector flow mapping intracardiac flow dynamic in mitral valve regurgitation

**DOI:** 10.3389/fcvm.2023.1047244

**Published:** 2023-03-24

**Authors:** Nicola Riccardo Pugliese, Andrea Colli, Giosuè Falcetta, Lavinia Del Punta, Carlo Puccinelli, Alessandro Fiocco, Anna Sonia Petronio, Stefano Taddei, Stefano Masi, Laura Besola

**Affiliations:** ^1^ Department of Clinical and Experimental Medicine, University of Pisa, Pisa, Italy; ^2^ Department of Surgical, Medical and Molecular Pathology and Critical Care Medicine, University of Pisa, Pisa, Italy

**Keywords:** intracardiac flow dynamic, mitral valve disease (MV disease), vector flow mapping (VFM), TMVR, TEER

## Abstract

**Objectives:**

The present study aims to assess and describe the intracardiac blood flow dynamic in patients with mitral regurgitation (MR), repaired mitral valves (MV) and mitral valve prostheses using vector flow mapping (VFM).

**Methods:**

Patients with different MV pathologies and MV disease treatments were analysed. All patients underwent 2D transthoracic echocardiography, and images for flow visualization were acquired in VFM mode in an apical three-chamber view and four-chamber view. Vectors and vortices were qualitatively analyzed.

**Results:**

thirty-two (32) patients underwent 2D transthoracic echocardiography (TTE) with VFM analysis. We evaluated intracardiac flow dynamics in 3 healthy subjects, 10 patients with MR (5 degenerative, 5 functional), 4 patients who underwent MV repair, 5 who underwent MV replacement (3 biological, 2 mechanical), 2 surgically implanted transcatheter heart valve (THV), 2 transcatheter edge-to-edge MV repair with MitraClip (TEER), 3 transcatheter MV replacement (TMVR) and 3 transapical off-pump MV repair with NeoChord implantation. Blood flow patterns are significantly altered in patients with MV disease and MV repair compared to control patients. MV repair is superior to replacement in restoring more physiologicalpatterns, while TMVR reproducesan intraventricular flowcloser to normal than surgical MVR and TEER.

**Conclusions:**

Intracardiac flow patterns can be clearly defined using VFM. Restoration of a physiological blood flow pattern inside the LV directly depends on the procedure used to address MV disease.

## Introduction

The study of intracardiac flow patterns has been initially performed using cardiac magnetic resonance (1–4). More recently, vector flow mapping (VFM), which is based on colour Doppler blood flow mapping and wall speckle tracking, has been used to describe the dynamic of blood flow patterns in mitral valve (MV) disease (5–7). Blood flows in the cardiac chambers create vortices that maintain the potential energy and optimize the cardiac workload and efficiency (8). Turbulent and not organized flow patterns are not efficient and increment the dissipation of potential energy deteriorating the ventricular function (9–12). Several studies demonstrated that surgical mitral valve repair (MVRe) is associated with better survival and left ventricular (LV) function improvement when compared to other treatments (surgical valve replacement, transcatheter valve repair or replacement) (13). This difference might be explained by a more physiological restoration of the intracardiac blood flow patterns after surgical repair, which translates into a reduced LV workload. Previously, Nakashima and Akiyama used VFM to flow patterns and energy dynamics of the intraventricular vortices in patients who underwent mitral valve surgery (6, 7). These studies show that flow dynamic is usually altered compared to healthy individuals independently from the type of treatment used to address the mitral valve disease; however mitral repair provided the most similar pattern to healthy individuals. Previous studies compared the intracardiac flow patterns and LV efficiency after surgical MVRe and MV replacement (MVR), confirming that repair usually preserves the normal LV vortices better than replacement (6, 9). However, no descriptive data are available regarding new techniques,such as transcatheter procedures, which are now taking over the scene of MV procedures, particularly in patients who might benefit the most from restoring the most physiologic flow pattern.

The present article aims to systematically describe the intracardiac blood flow patterns of the entire spectrum of MV regurgitation disease and the possible alternative techniques to address it.

## Methods

Between June 1st, 2021 and January 31st, 2022, healthy subjects, patients with functional and degenerative MV disease and patients who underwent MV treatments at our Centre, either surgical or transcatheter, were included in the study. The reason for MV surgical or transcatheter MV treatment was isolated MR, either degenerative or functional. All patients who underwent MVRe or MVR presented with degenerative disease, while patients undergoing transcatheter procedures presented variably with degenerative or functional disease. No other procedures were performed. Patients with inadequate acoustic window and/or atrial fibrillation at the time of image acquisition were excluded from the study. All patients signed a dedicated informed consent before being included in the present study that was approved by the local Ethical Committee. At 1 month after surgery all patients underwent dedicated 2D transthoracic echocardiography. None of the patients had TTE both before and after surgery. Intracardiac flow images were recorded using a 5-MHz matrix array single crystal technology ultrasound transducer (Hitachi Medical Systems, Tokyo, Japan). Images for flow visualisation were acquired in VFM mode in an apical three-chamber view and four-chamber view. All images were acquired in three consecutive cardiac cycles with a target frame rate set inthe range of 20–25 frames/s. The color Nyquist limit was set sufficiently high to mitigate aliasing phenomena, and automatic self-aliasing was used as well. The Doppler signal provided the axial component of the blood velocity, while the time-varying position of the boundaries (LV walls) was obtained from 2D STE. The offline analysis was computed with dedicated software (DAS-RS1 5.0; Hitachi Medical Systems, Tokyo, Japan). The cardiac cycles were determined according to the valve openings and closings with the synchronous ECG: T wave defined the end of the contraction, while the R peak of the QRS complex set the end of the diastolic phase. The LV endocardial border was traced in the first frame image, and the software automatically tracked the endocardial border throughout the cardiac cycle. After determining the ROI, qualitative images (velocity vectors and streamlines) were displayed in 2D for each frame of the cine loop image.Energy loss expresses the amount of energy dissipated as heat in the LV by viscous friction in turbulent blood during the process of relaxation (diastole) and contraction (systole). Since turbulent flow results in an irreversible loss of the total fluid energy, the higher the EL, the greater the LV inefficiency. After tracing the ROI of the LV cavity, a sample line was placed at the level of the mitral annulus (in the apical four-chamber and long-axis view) and in the LV outflow tract(in the apical long-axis view) to acquire the time-flow curves of the diastolic and systolic period, respectively. Therefore, EL is quantified as the sum of the square of the difference between adjacent velocity vectors:

EL=∑i,j∫12μ(∂ui∂xj+∂uj∂xi)2∂vEL=∑i,j∫12μ∂ui∂xj+∂uj∂xi2∂v

where µ is the coefficient of blood viscosity (µ blood = 4.0 × 10 −3 Newton·s·m −2), u and x are the velocity vector components, i and j are the coordinates of the Cartesian system built on a 2D vector field, and v is the vector velocity. EL represents the rate at which energy is expended in a 2D system; hence, it is measured in watts/m: W/m = Joule/(m·s). According to the formula, EL increases at points where the size and direction of velocity vectors change. EL measurements were estimated from an apical three-chamber view.

## Results

Thirty-two (32) patients were evaluated. We included 3 healthy individuals to describe the normal intracardiac flow patterns. Ten (10) patients presented severe mitral regurgitation (MR) (5 degenerative-DMR, 5 functional-FMR), 4 patients underwent MVRe (3 with annuloplasty ring and posterior leaflet resection and 1 with ring and artificial polytetrafluoroethylene chordae), 5 patients had a surgical mitral prosthesis (3 biological, 2 mechanical all in antianatomic orientation), 2 patients had a Sapien 3 transcatheter heart valve (Edwards Lifesciences, Irvine, CA, USA) surgically implanted through the left atrium with anterior mitral leaflet resection, 2 patients underwent transcatheter edge-to-edge mitral valve repair (TEER) with a MitraClip (Abbott Vascular, Plymouth, MN, USA), 3 patients had a transcatheter mitral valve replacement (TMVR) with a Tendyne prosthesis (Abbott Vascular, Plymouth, MN, USA) and 3 patients underwent transapical off-pump mitral valve repair with NeoChord implantation (NeoChord, Inc., St. Louis Park, MN, USA).

Out of the 5 patients with DMR 2 had an extensive posterior multi-scallop disease (Barlow-type), while the other 3 presented with fibroelastic deficiency and less extensive leaflet involvement (prolapse or flail of one scallop). Patients with FMR had underlying ischemic cardiomyopathy in 2 cases while primitive dilative cardiomyopathy in 3 cases.

All mechanical valves were bileaflet Regent prostheses (Abbott Vascular, Plymouth, MN, USA), while all implanted biological valves were Mosaic porcine prostheses (Medtronic, Minneapolis, MN, USA). All patients had their subvalvular apparatus preserved, and in case of mechanical replacement, the prosthesis had an antianatomical orientation.

In all cases of MVRe, a semi-rigid Simulus annuloplasty ring was used (Medtronic, Minneapolis, MN, USA).

Both patients who underwent TEER primarily presented with ischemic cardiomyopathy and showeda residual mild-to-moderate MR after the procedure. TMVR with low-profile Tendyne prosthesis was performed in patients with FMR due to dilative or ischemic cardiomyopathy. All patients treated with Sapien 3 prosthesis presented severe mitral annular calcification (MAC) and underwent concomitant anterior mitral leaflet resection (AML) to prevent the occurrence of potential left ventricular outflow tract (LVOT) obstruction.

The three patients who underwent Neochord procedure presented degenerative MR involving the posterior mitral leaflet (PML) with preserved LV function. One of them previously underwent mitral valve repair receiving isolated annular ring implantation.

Complete transthoracic echocardiography data and procedural details of the patients which were selcted as images and video examples for each condition are reported in Table 1.

**Table 1:** Ultrasound evaluation.

Variable	Normal MV	Degenerative MR	Functional MR	MV repair	TEER	MV replacement	MV replacement	TMVR	Sapien 3 in MAC	NeoChord TM Device
**Gender**	Female	Male	Female	Male	Male	Male	Female	Female	Female	Male
**Age, years**	54	75	73	60	79	44	71	72	70	49
**Technical procedure**	–	–	–	1 pair of artificial chord for P2 + Medtronic Simulus 36 annuloplasty ring	Transcatheter mitral edge-to-edge with 2 MitraClips	Medtronic Mosaic 33	Corcym Carbomedics 29	Transcatheter Tendyne LP 33S	Surgical Sapien 3 29	Transapical off-pump implantation of 3 pairs of Neochords for P2
**Conventional echocardiography**										
LVMi, g/m^2^	75	110	122	100	159	136	90	120	113	150
LVEDV, mL	80	145	149	148	198	185	110	138	124	181
LV Ejection fraction, %	60	60	45	60	42	43	59	45	67	60
Mitral E wave, cm/s	80	110*	150	87	105	110	135	100	125	84
Average e’, cm/s	11	16	6	9	7	8	7	7	6	10
Average E/e’	7	7	25	10	15	14	19	14	21	11
LAVi, mL/m^2^	19	38	49	42	52	41	40	42	47	32
TAPSE, mm	21	20	18	16	19	15	17	17	17	17
Systolic PAP, mmHg	20	35	59	35	38	40	35	38	40	22
**Vector Flow Mapping**										
Energy loss, J/m·s*	0.36	0.51	0.65	0.39	0.67	0.48	0.55	0.47	0.74	0.41

EDV, end-diastolic volume; LAVi, left atrial volume index; LV, left ventricle; LVMi, left ventricle mass index; PAP, pulmonary artery pressure; TAPSE, tricuspid annular plane systolic excursion; MV, mitral valve; MR, mitral regurgitation; TEER, transcatheter edge-to-edge repair; TMVR, transcatheter mitral valve replacement; MAC, mitral annulus calcification.

* Expressed as the mean energy loss of three complete cardiac cycles.

### Flow analysis

**Figure 1:**
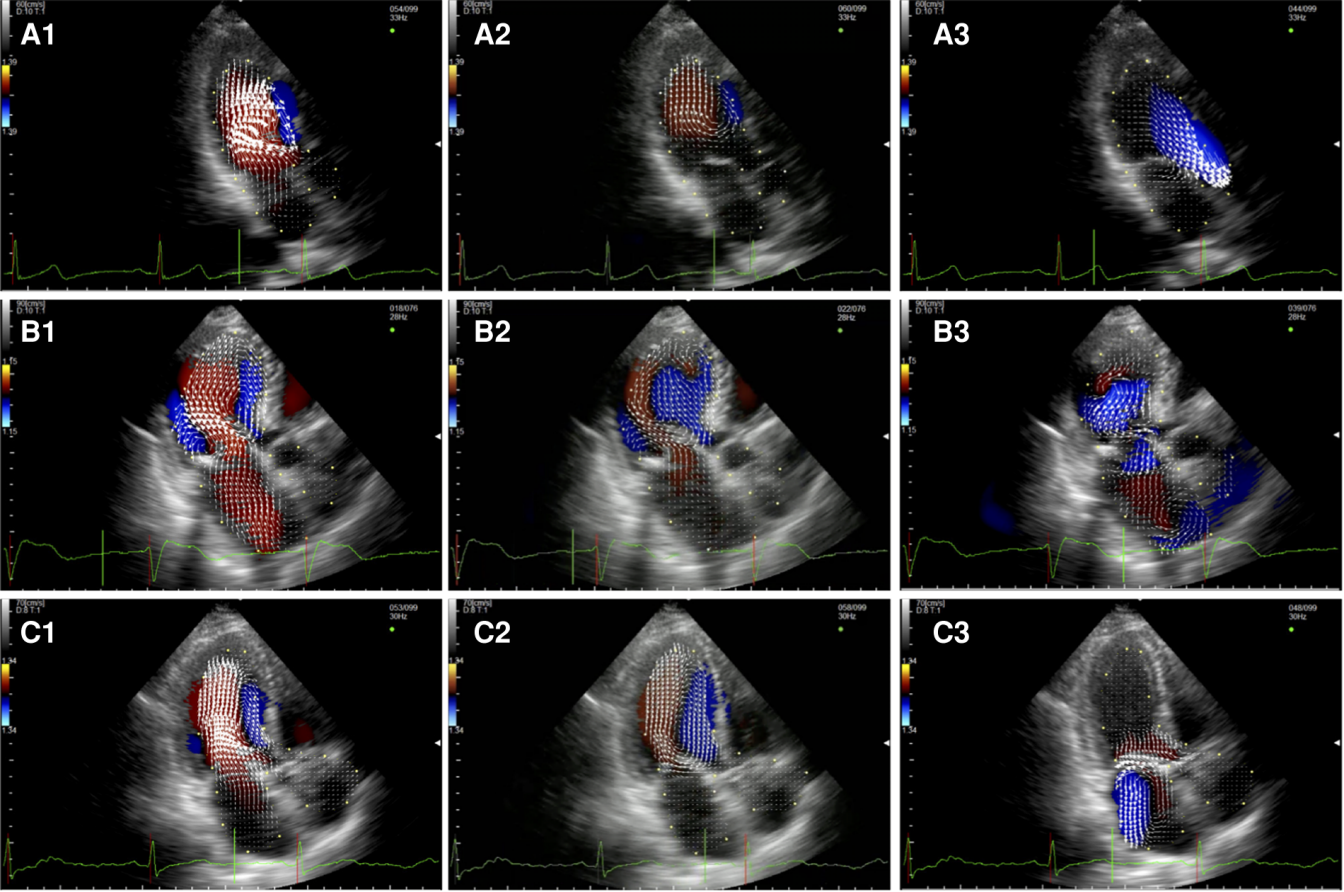
Apical long-axis view. Intracardiac flow vectors in early diastole (**A1–C1**) and late diastole (**A2–C2**) for control patient, patient with DMR and patient with FMR respectively; intracardiac flow vectors in mid-systole for control patient (**A3**), patient with DMR (**B3**) and patient with FMR (**C3**).

In the healthy controls (Figure 1, panel A1,A2,A3, Supplementary Video S1), blood enters the LV cavity through the MV smoothly, creating two vortices that move in opposite directions, a main clockwise vortex beneath the AML and a smaller counterclockwise vortex beneath the PML. While the posterior vortex dissipates quickly, the anterior vortex keeps getting bigger during diastole moving downstream and pushing the blood flow towards the posterior wall of the LV and then redirecting the flow towards the LVOT during systole. In systole, hemodynamic forces are directed mainly along the left ventricle longitudinal axis, from the apex to the LVOT, without significant vortices (i.e., vortices that persist for at least two consecutive frames).

In patients with DMR (Figure 1, panel B1,B2,B3, Supplementary Video S2),the diastolic flow pattern composed ofthe two LV vortices is preserved. However, during late diastole and early systole, the anterior vortex does not direct the flow toward the LVOT because the hemodynamic forces are directed from the apex to both the LVOT and the left atrium due to the posterior leaflet prolapse, leading to the formation of multiple vortices proximal to the MV (mainly with a clockwise rotation). In FMR (Figure 1 panel C1,C2,C3, Supplementary Video S3), we observed only one clockwise vortex distal to the AML during early filling. In systole, because of PML tethering causing MV malcoaptation, hemodynamic forces are directed from the apex both to the LVOT and to the left atrium leading to the formation of a counterclockwise vortex proximal to the MV.

**Figure 2:**
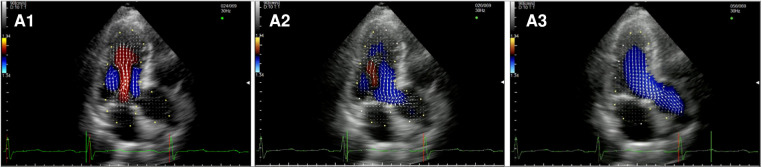
Apical long-axis view. Intracardiac flow vectors in mid-to-late diastole (**A1**,**A2**) and mid-systole (A3) after surgical mitral valve repair neochords and annuloplasty ring.

After MVRe, the intracavitary blood flow pattern is mostly restored (Figure 2, Supplementary Video S4). Whether a triangular resection alone or neochords implantation was combined with the annuloplasty ring the formation of two vortices with the movement of the blood flow toward the posterior wall of the LV could be observed, optimizing LV forces and minimizing the turbulence in the LVOT during systole. The only difference with healthy control was the slightly longer persistence of the posterior vortex during diastole.

**Figure 3:**
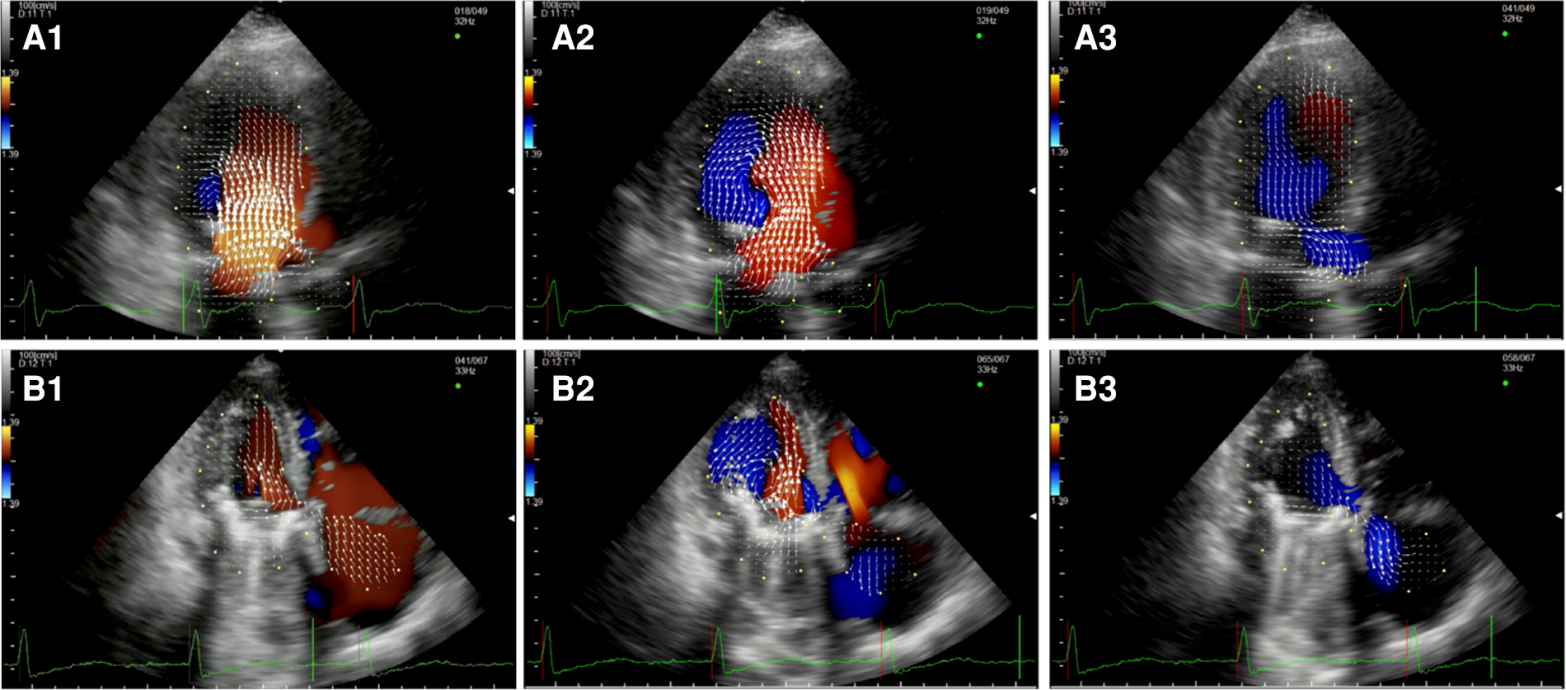
Apical long-axis view. Intracardiac flow vectors in early diastole (**A1,B1**) and late diastole (**A2,B2**) after mitral valve replacement with bioprosthesis and mechanical prosthesis (antianatomicalorientation) respectively; intracardiac flow vectors in mid-systole (**A3,B3**) after mitral valve replacement with bioprosthesis and mechanical prosthesis (antianatomicalorientation) respectively.

After MVR with bioprostehsis (Figure 3, panel A1,A2,A3, Supplementary Video S5), we can observe in diastole only one vortex distal to the bioprosthetic valve with a counterclockwise rotation (opposite to the healthy control). The vortex occupies the center of the LV cavity to redirect blood toward the LVOT. In systole, we observed intraventricular vortices probably related to the LV systolic dysfunction. Moreover, it is possible to notice a vortex rotating clockwise within the struts of the bioprosthesis. Similarly, in patients with a mechanical prosthesis in antianatomical orientation (Figure 3, panel B1,B2,B3, Supplementary Video S6), during diastole, there is a major counterclockwise vortex in the LV mid cavity and a smaller clockwise one that disappears quickly. The main vortex redirects the flow towards the LVOT. In this group, turbulence during systole is less evident.

**Figure 4:**
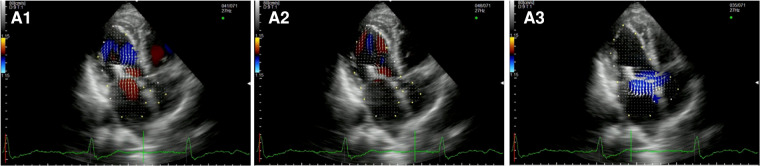
Apical long-axis view. Intracardiac flow vectors in early and late diastole (**A1,A2**) and mid-systole (**A3**) after transcatheter mitral valve repair with MitraClip (residual mild-moderate MR).

After TEER (Figure 4, Supplementary Video S7), during diastole, we observe multiple vortices without forming the typical main clockwise vortex occupying the center of the cavity. In systole, hemodynamic forces are partially restored along the LV longitudinal axis, from the apex to the LVOT, with an incomplete pair of counterrotating vortices in the left atrium due to the residual MR.

**Figure 5:**
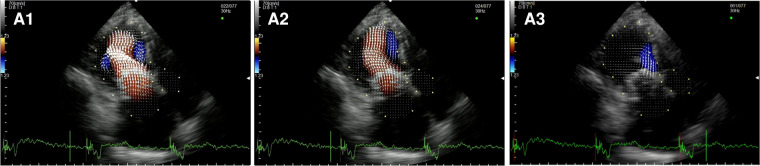
Apical long-axis view. Intracardiac flow vectors in early and late diastole (**A1,A2**) and mid-systole (**A3)** after transcatheter mitral valve replacement with Tendyne system.

The three patients who underwent TMVR presented a nearly normal flow pattern (Figure 5, Supplementary Video S8). During early filling, we observed the formation of the typical two counterrotating vortices, with the major one below the AML pushing back the blood flow and redirecting it towards the LVOT during mid-systole.

**Figure 6:**
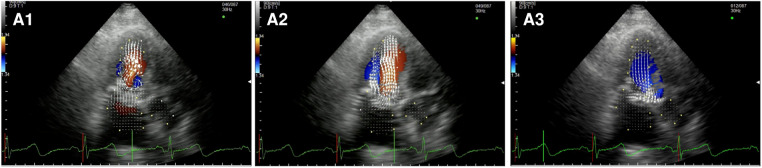
Apical long-axis view. Intracardiac flow vectors in early and late diastole (**A1,A2**) and mid-systole (**A3**) after surgical implantation of a Sapien 3 valve in mitral annular calcification (MAC).

In the two subjects who underwent surgical implantation of a Sapien 3 THV (Figure 6, Supplementary Video S9), there was a pair of counterrotating vortices during diastole. Still, the main vortex had a counterclockwise rotation (opposite to the healthy control). In systole, hemodynamic forces were directed mainly along the left ventricle longitudinal axis, from the apex to the LVOT. Multiple secondary vortices occur throughout the cardiac cycle distal and proximal to the bioprosthesis.

**Figure 7:**
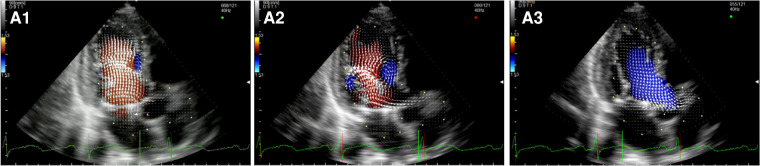
Apical long-axis view. Intracardiac flow vectors in early and late diastole (**A1,A2**) and mid-systole (**A3**) after transapical off-pump mitral valve repair with NeoChord TM device.

After Neochord Procedure (Figure 7, Supplementary Video S10), we observed a flow pattern that was similar to healthy subjects and patients who underwent surgical repair with a larger clockwise vortex below the anterior leaflet and the smaller counterclockwise one under the posterior leaflet during early diastole. Again we observed the displacement of the flow vectors towards the apical region during the later phases of the diastole and only mildly turbulent systolic flow in the LVOT.

**Figure 8:**
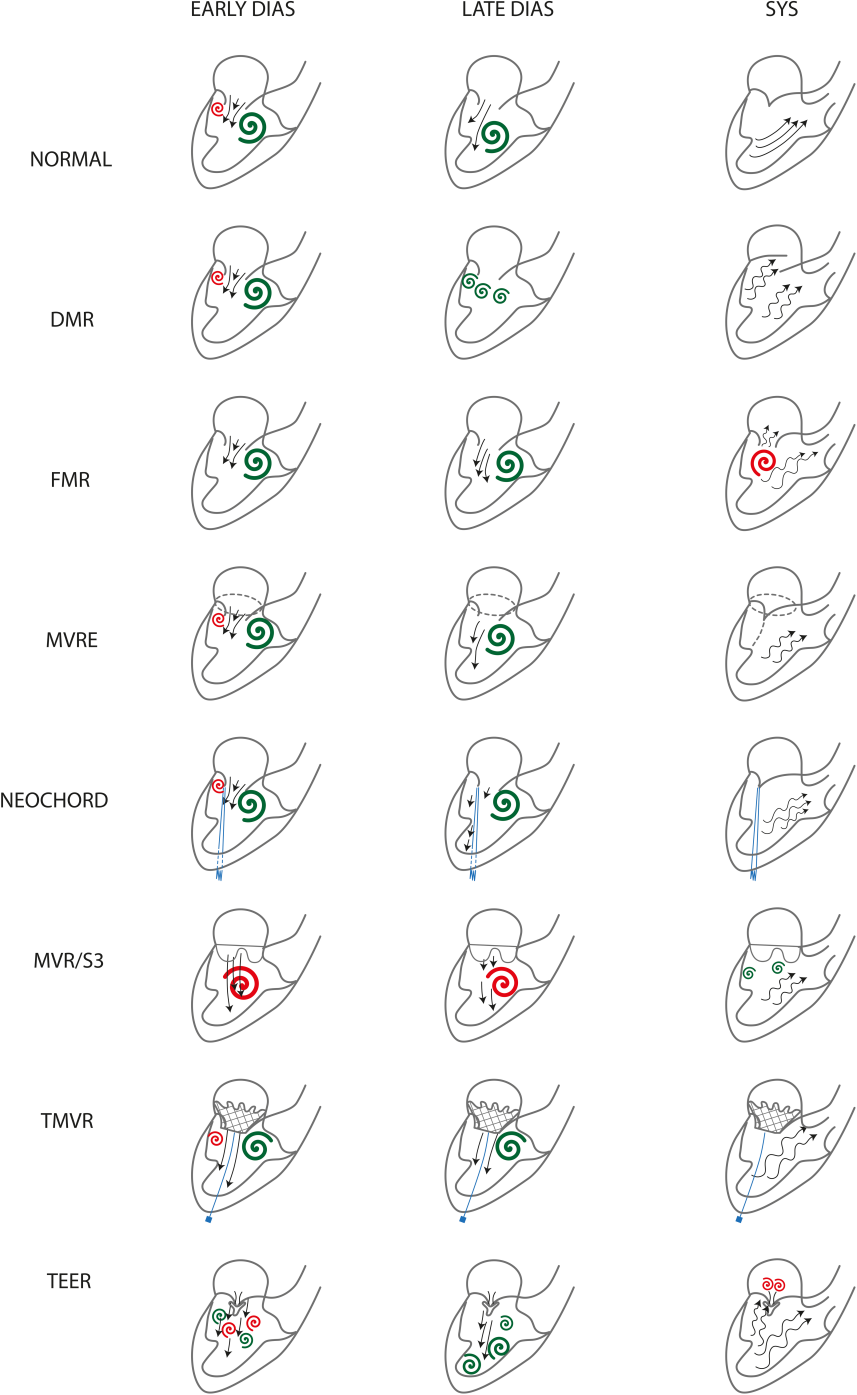
Flow patterns graphical representation. Clockwise vortices are green, counterclockwiseones are red. DMR, degenerative mitral regurgitation; FMR, functional mitral valve regurgitation; MVRe, mitral valve repair; Neochord, transapical off-pump mitral valve repair witrhNeoChord implantation; MVR, mitral valve replacement; S3, Valve in MAC with Edwards Sapien 3; TMVR, transcatheter mitral valve replacement; TEER, transcatheter edge-to-edge mitral valve repair.

Graphical representation of our findings is also shown in Figure 8.

More images and videos depicting the intracavitary vortices and the enery loss loops are available as Supplemenary Material.

## Discussion

There is increasing interest in studying intracardiac blood flow patterns to clarify the physiopathological mechanisms at the base of different cardiac conditions, and various imaging techniques have been developed over the last few years (8, 14). Apart from 4D flow MRI, which enables an extremely accurate 3D evaluation of vortex flow patterns and LV workload through EL quantification on the base of a single cardiac cycle (1, 15), echocardiography has been reliable in visualizing intracardiac flows (16, 17). In particular, echocardiography VFM, based on color-Doppler and speckle-tracking techniques, was developed (18, 19) and has already been used to analyseflows after cardiac surgery (6, 7) in particular in the field of mitral valve surgery. Since the late 1980s,MVRe has been superior to MVR in preserving LV function (13). One possible explanation of this phenomenon is the preservation of the subvalvular apparatus that causes a reduction of the LV radius and, therefore, the LV wall stress (20, 21). However, this explanation does not entirely justify the dramatic difference in LV function preservation. Restoring normal intracardiac LV flow patterns observed mainly after MVRe may helppreservethe kinetic energy momentum reducing LV workload and shear stress as previously demonstrated (8).

This is the first comprehensive description of LV flow patterns in MV disease using VFM. The main findings of our descriptive analysis are that intracardiac blood flow patterns are restored after MVRe independently from the repair technique, MVR (both with biological and mechanical prostheses in antianatomical orientation) is affected by the persistence of altered blood flows, TEER completely alters LV vortices and TMVR with Tendyne valve has a similar effect to MVRe on flow patterns restoration (Figure 8).

Our findings in healthy subjects are largely comparable to those reported in the literature. The physiological flow pattern was also confirmed in our healthy subjects. However, we did not observe prolonged persistence of vortices during early systole as elsewhere described (22, 23). This difference might be explained because we considered as relevant only vortices persisting longer than two consecutive frames. However, based on the small study population, it is possible that more significant vortices were not observed because they were not included in the imaging plane. A larger study could solve this issue, albeit the diastolic pattern was largely comparable with that reported by other authors. In the presence of FMR, flow patterns were altered and presented some similarities with those described by other authors. Indeed we observed only one large vortex that moved from the MV to the mid cavity with an initial clockwise rotation and a late counterclockwise movement. Pilla et al. (24) found the presence of two vortices below the MV leaflets, with the anterior one bigger than the posterior one and a later single mid-cavity vortex. Differently from Pilla, who analysed suine models in which a posterolateral infarction was induced, all our patients did not have specific regional wall abnormalities, which could explain these small differences in intraventricular flows.

The conservation of the kinetic energy is enabled by the presence of vortices, particularly the anterior clockwise vortex, which appears beneath the AML. The formation of this vortex is guaranteed by the higher length of the AML comparedto the PML and the apical direction of the flow through the MV, as previously described by Pedrizzetti et al. (25).

MVRe preserves this difference in length since surgeons usually try to maintain the physiological 2/3:1/3 ratio between AML and PML surface mostly reducing the PML height to prevent an anteriorly displaced coaptation line that would increase the risk of AML systolic anterior motion. Our observations were consistent with these findings since in all patients who underwent MVRe we could appreciate the formation of a large anterior vortex that pushed the blood flow towards the posterior LV wall helping the flow entering the LVOT. In our experience, similarly to what was reported by other groups (2, 7), the type of repair did not affect physiological vortex formation; we only observed a slightly prolonged persistence of the posterior counterclockwise vortex in patients who received chordal implantation. However, this observation did not determine any significant increase of EL during the cardiac cycle or the formation of turbulent patterns in the LVOT. Morichi et al. (2), using 4D flow MRI, studied the effect of different types of annuloplasty rings and bands on intracardiac flow patterns. They observed a more preserved pattern when a flexible band or ring was used compared to semi-rigid bands or rings. In our series, only semi-rigid rings were implanted. In all these patients, the flow pattern was similar to healthy controls. The absence in our series of the flow alteration observed by Morichi et al. in semi-rigid rings might be explained by the large size of the rings we implanted. A smaller orifice area is associated with a larger posterior vortex which pushes the flow toward the anterior wall opposite to the healthy control. The desired effect of ring annuloplasty is to reduce the AP diameter without excessive reduction of the MV orifice area. For this reason, the implanted ring should be onesize greater than the actual measured AML area. Moreover, Morichi's group supposed that the increased EL observed for semi-rigid rings was also determined by the restrictive effect of the ring on the LVOT. This condition does not apply to the Simulus ring,whichhas a very soft and adaptable anterior section. Interestingly Neochord Procedure was associated with similar findings to standard surgical repair, confirming the restoration of pseudonormal flow patterns independently from the presence of the annuloplasty ring and supporting the hypothesis of future positive remodelling of the LV also when the MV ring is not addressed.

Also, our observations confirm that MVR does not restore physiological flow patterns (6, 9). Other groups found a difference between biological prostheses and anatomically oriented mechanical prostheses; only the latter provided two counterrotating vortices, while the former determined the creation of a single counterclockwise vortex that pushed the blood flow towards the anterospetal wall, then to the apex and finally to the posterior wall. In our experience, we could only examine biological prostheses and antianatomically oriented mechanical valves, and in both cases, a single large counterclockwise vortex was observed. Other groups had similar findings regarding mechanical valves in antianatomical orientation. This difference might be explained by how the blood enters the LV cavity. The sutures used to implant a MV prosthesis follow the saddle shape of the MV annulus orienting the valve oblique with respect to the posterior annulus level. Indeed the anterior portion of the sewing ring tends to be positioned higher thanthe posterior aspect on the mitral annulus plane; this anteriorly tilted angle creates an altered entrance angle for the blood, which is naturally directed towards the anteroseptal wall creating the large counterclockwise vortex. A similar condition is observed for the surgically implanted THV Sapien 3 valve. When deployed within the MV annulus, the THV slides posteriorly, remaining higher on the anterior aspect of the annulus anddirecting the flow anteriorly inside the LV chamber. To prevent paravalvular leaks, the surgeon manually sutures an adjunctive Teflon skirt on the atrial portion of the THV stent to seal the stent to the periannular area (26, 27). It is possible that trimming this skirt to slide the THV higher on the posterior annulus might prevent the anterior tilting providing a more apical-directed LV flow and enabling the formation of the physiological double vortices. Our observations on TMVR support this theory. Because of the apical pad that anchors the THV to the LV wall and the particular shape of the valve stent and atrial skirt, the prosthesis is forced to a more coplanar position to the MV annulus. Indeed the flow pattern observed for Tendyne is very close to a normal subject.

Finally, TEER completely disrupts the LV physiological vortices creating turbulent flow during diastole and systole. There are no reports about intracardiac patterns after TEER, and ours are the first descriptive findings. We hypothesize that this flow disarray is due to the combination of two elements. First, the creation of a double orifice valve with the major axis oriented at 90° with respect to the LV major axis as it is observed for mechanical valves in antianatomic orientation; second, the presence of a rigid element (the clip) attached to the leaflets that prevents the normal swirling of the blood flow around the tips of the leaflets. This finding should be carefully evaluated since TEER is frequently indicated in patients with reduced LV function, who might be the ones that might benefit the most from the restoration of normal LV flow patterns reducing LV workload and enhancing LV function recovery.

Three subjects of the study population presented HFrEF, one of them underwent TEER, another received a surgical bioprosthesis, and the third one had TMVR. In all cases, LV impairment was due to global hypokinesia with no regional wall motion abnormalities and with PML tethering. HFrEF was associated with altered intracardiac flow patterns (28) in the presence of apically displaced vortices. This is consistent with our findings in the HFrEFpatients who underwent MVR with a bioprosthesis; however, no such observations were made after TEER and TMVR. Unfortunately,TEER completely disrupts intracardiac flow patterns making it impossible to evaluate any possible effect of LV function of vortex formation. Interestingly the patient who had TMVR seemed not to be affected by HFrEF-related abnormalities and presented an almost complete restoration of a normal flow. This might be explained by a fully reversible myocardial impairment that responded well to volume and pressure overload resolution. Such inferences should be confirmed by advanced myocardial function analysis, as LV wall strain.

The main limitation of the present research is the small, heterogeneous sample size. However, this is a pivotal study analyzing all currently available MV treatments. It couldopen up the field of VFM analysis of MV surgery to define the optimal strategy to address MV disease.

Moreover, VFM evaluation is based on echocardiography which is operator dependent. All studies and post-processing analyses were performed by the same operator. More reproducible imaging techniques exist, such as cardiac MR, butthey are more expensive, present several limitationsand are less tolerated by the patient.

Finally, this is a 2D-TTE study. As demonstrated by the wide flow disruption after TEER in all 3 dimensions of the space, it is plausible that some of the vortices might have been missed because out of the imaging plane. 3D-TTEmight overcome this problem and shed new light on intracardiac flow mapping.

In conclusion, our findings confirm that intracardiac flow patterns are altered in patients with mitral disease and that MVRe is superior to MVR in restoring normal patterns. Based on our observations, TMVR might represent a better strategy than TEER to optimize LV function in the mid and long-term, thanks to its nearly normal flow patterns. Finally, better angulation of prosthetic valves and anatomical orientation can lead to more physiological intracardiac flow dynamics.

Our findings, even if qualitative and limited to a small study population, suggest that the treatment used to correct MR have an impact on postoperative intracardiac flow patterns. In the surgical community is a shared knowledge that MVRe is preferable over MVR because of better long-term outcomes. Similarly, TEER is associated with improved survival and symptoms only in selected patients. We believe that further studies of postprocedural intracardiac flow patterns can explain these differences supporting one therapy over another because more efficient in preserving or restoring physiological flow patterns.

## Data Availability

The raw data supporting the conclusions of this article will be made available by the authors, without undue reservation.

## Supplementary material

Supplementary Figure S1 Apical long-axis view. Intracardiac vortices in early diastole (A) and mid systole (B) for control patient.

Supplementary Figure S2 Apical long-axis view. Intracardiac vortices in early diastole (A) and mid systole (B) for patient with DMR.

Supplementary Figure S3 Apical long-axis view. Intracardiac vortices in early diastole (A) and mid systole (B) for patient with FMR.

Supplementary Figure S4 Apical long-axis view. Intracardiac vortices in early diastole (A) and mid-systole (B) after surgical mitral valve repair with neochords and annuloplasty ring.

Supplementary Figure S5 Apical long-axis view. Intracardiac vortices in early diastole (A) and mid-systole (B) after transcatheter mitral valve repair with MitraClip (residual mild-moderate MR).

Supplementary Figure S6 Apical long-axis view. Intracardiac vortices in early diastole (A) and mid systole (B) after mitral valve replacement with bioprosthesis.

Supplementary Figure S7 Apical long-axis view. Intracardiac vortices in early diastole (A) and mid systole (B) after mitral valve replacement with mechanical prosthesis.

Supplementary Figure S8 Apical long-axis view. Intracardiac vortices in early diastole (A) and mid-systole (B) after transcatheter mitral valve replacement with Tendyne system.

Supplementary Figure S9 Apical long-axis view. Intracardiac vortices in early diastole (A) and mid-systole (B) after surgical implantation of a Sapien 3 valve in mitral annular calcification (MAC).

Supplementary Figure S10 Apical long-axis view. Intracardiac vortices in early diastole (A) and mid-systole (B) after transapical off-pump mitral valve repair with NeoChord TM device.

Supplementary Video S1 Energy loss distribution during the cardiac cycle in the left ventricle in control patient.

Supplementary Video S2 Energy loss distribution during the cardiac cycle in the left ventricle in patient with degenerative mitral regurgitation.

Supplementary Video S3 Energy loss distribution during the cardiac cycle in patient with functional mitral regurgitation.

Supplementary Video S4 Energy loss distribution during the cardiac cycle after mitral valve repair with neochords and annuloplasty ring.

Supplementary Video S5 Energy loss distribution during the cardiac cycle after transcatheter mitral valve repair with MitraClip.

Supplementary Video S6 Energy loss distribution during the cardiac cycle after mitral valve replacement with bioprosthesis.

Supplementary Video S7 Energy loss distribution during the cardiac cycle after mitral valve replacement with mechanical prosthesis.

Supplementary Video S8 Energy loss distribution during the cardiac cycle after transcatheter mitral valve replacement with Tendyne system.

Supplementary Video S9 Energy loss distribution during the cardiac cycle after surgical implantation of a Sapien 3 valve in mitral annular calcification (MAC).

Supplementary Video S10 Energy loss distribution during the cardiac cycle after transapical off-pump mitral valve repair with NeoChord TM device.
