# Supplementary figures and images for: Flow dynamic assessment of native mitral valve, mitral valve repair and mitral valve replacement using vector flow mapping intracardiac flow dynamic in mitral valve regurgitation
Source: Front Cardiovasc Med. 2023 Mar 24;10:1047244. doi: 10.3389/fcvm.2023.1047244 (PMC10080047; doi:10.3389/fcvm.2023.1047244)

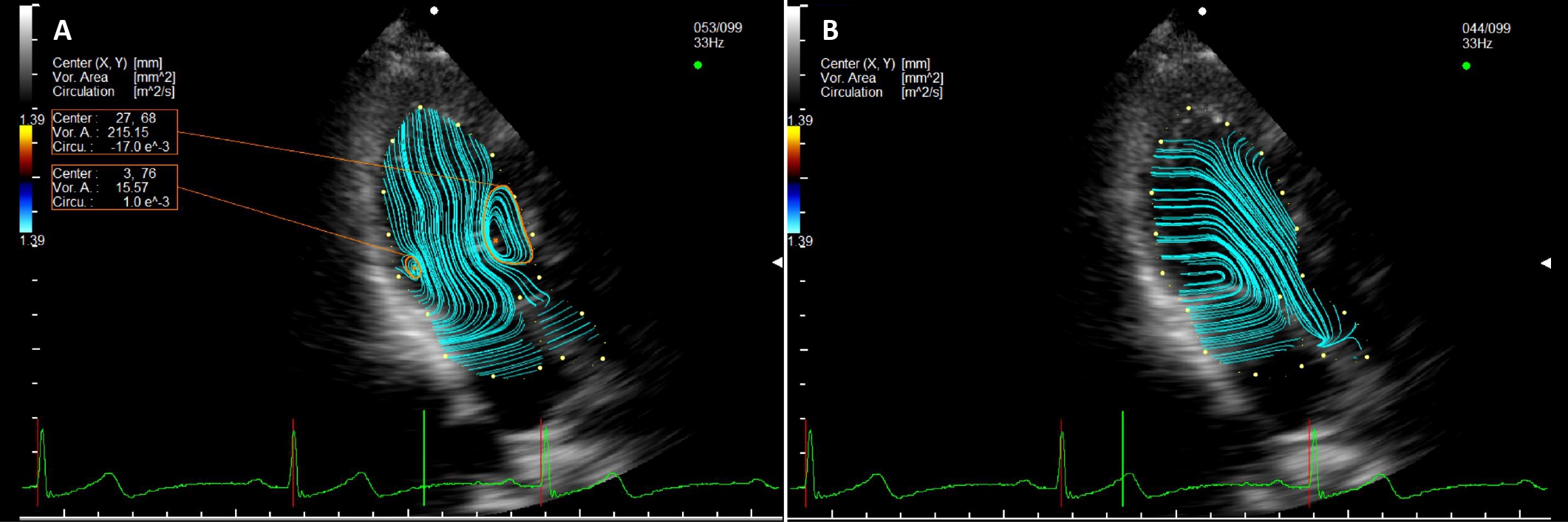

Supplement: Supplementary Figure S1 — Apical long-axis view. Intracardiac vortices in early diastole (A) and mid systole (B) for control patient. [file Image1.jpeg]

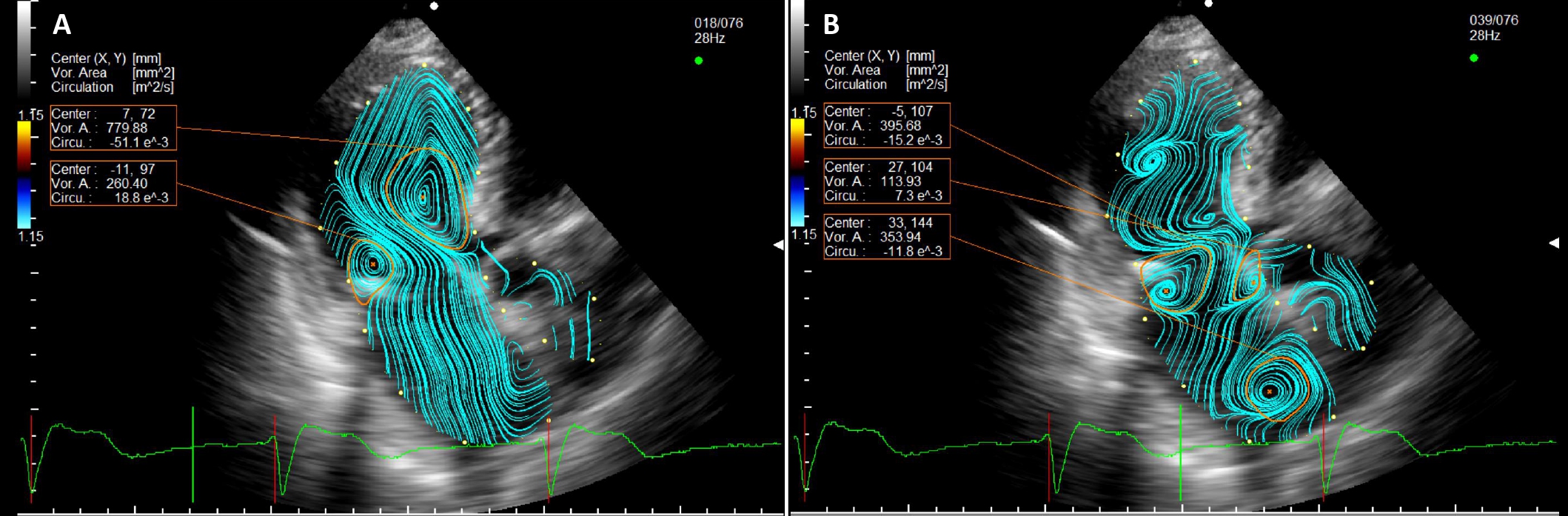

Supplement: Supplementary Figure S2 — Apical long-axis view. Intracardiac vortices in early diastole (A) and mid systole (B) for patient with DMR. [file Image2.jpeg]

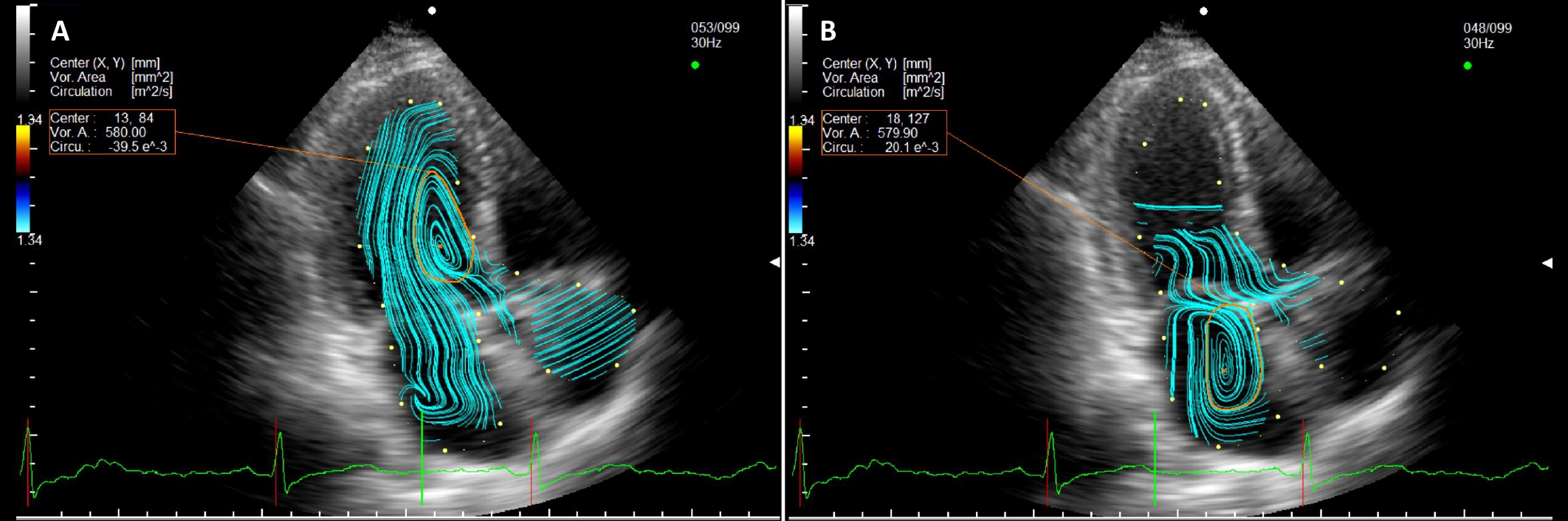

Supplement: Supplementary Figure S3 — Apical long-axis view. Intracardiac vortices in early diastole (A) and mid systole (B) for patient with FMR. [file Image3.jpeg]

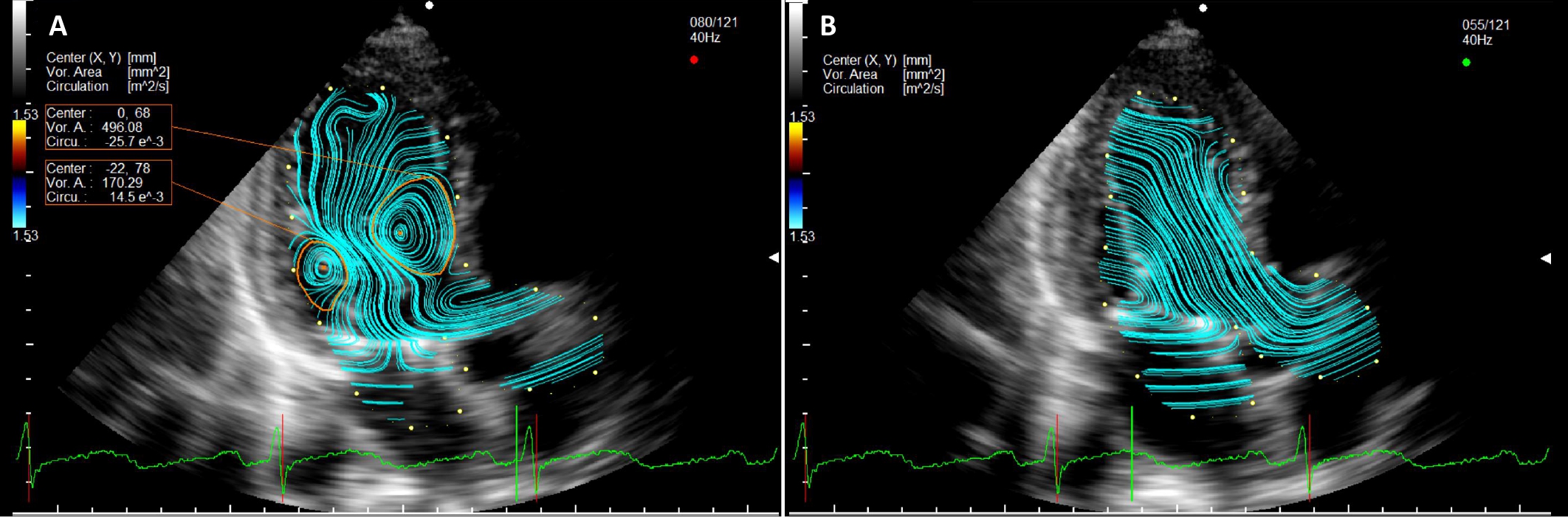

Supplement: Supplementary Figure S4 — Apical long-axis view. Intracardiac vortices in early diastole (A) and mid-systole (B) after surgical mitral valve repair with neochords and annuloplasty ring. [file Image4.jpeg]

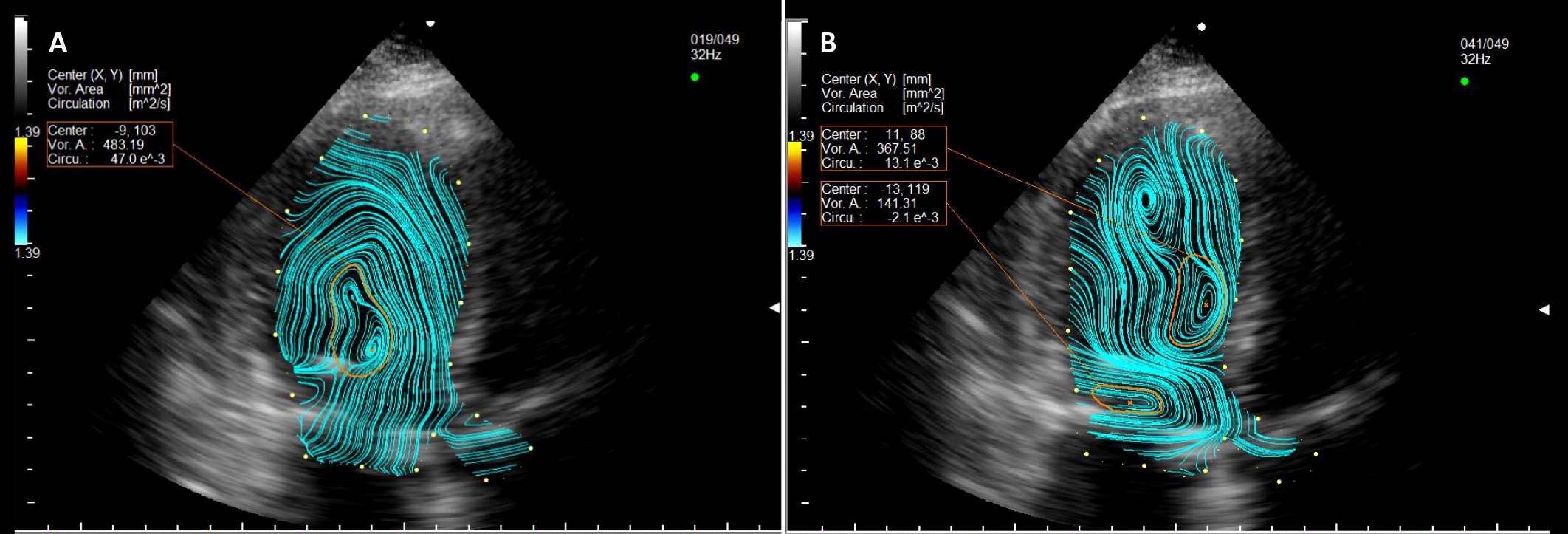

Supplement: Supplementary Figure S5 — Apical long-axis view. Intracardiac vortices in early diastole (A) and mid-systole (B) after transcatheter mitral valve repair with MitraClip (residual mild-moderate MR). [file Image5.jpeg]

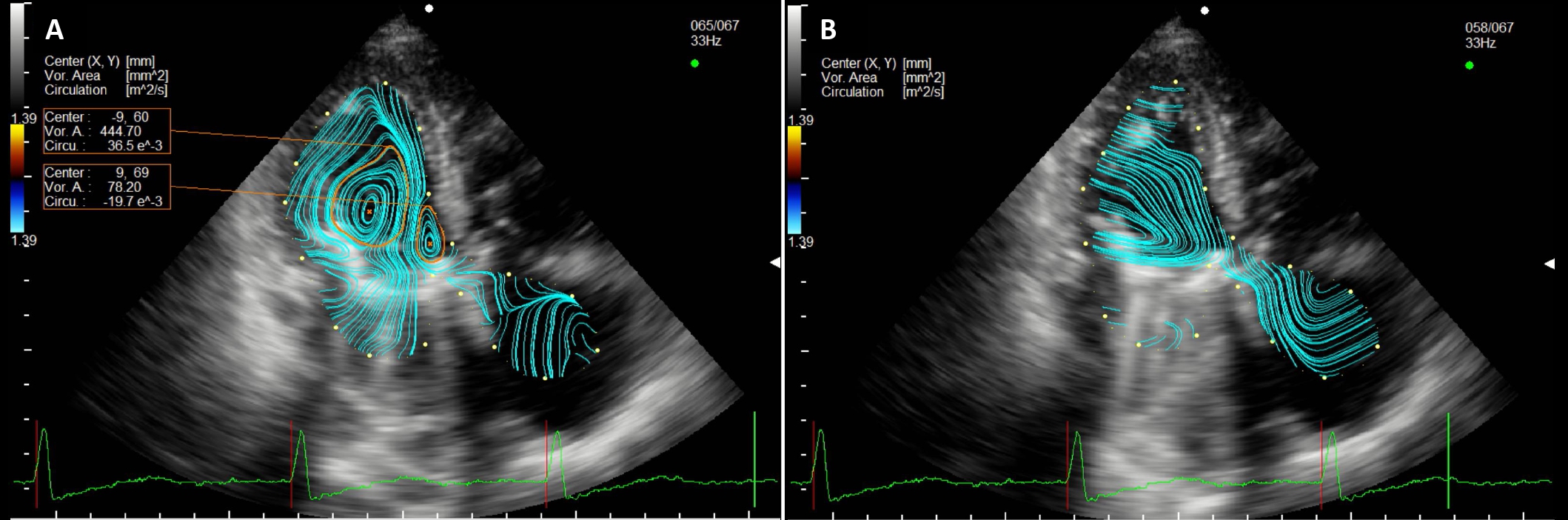

Supplement: Supplementary Figure S6 — Apical long-axis view. Intracardiac vortices in early diastole (A) and mid systole (B) after mitral valve replacement with bioprosthesis. [file Image6.jpeg]

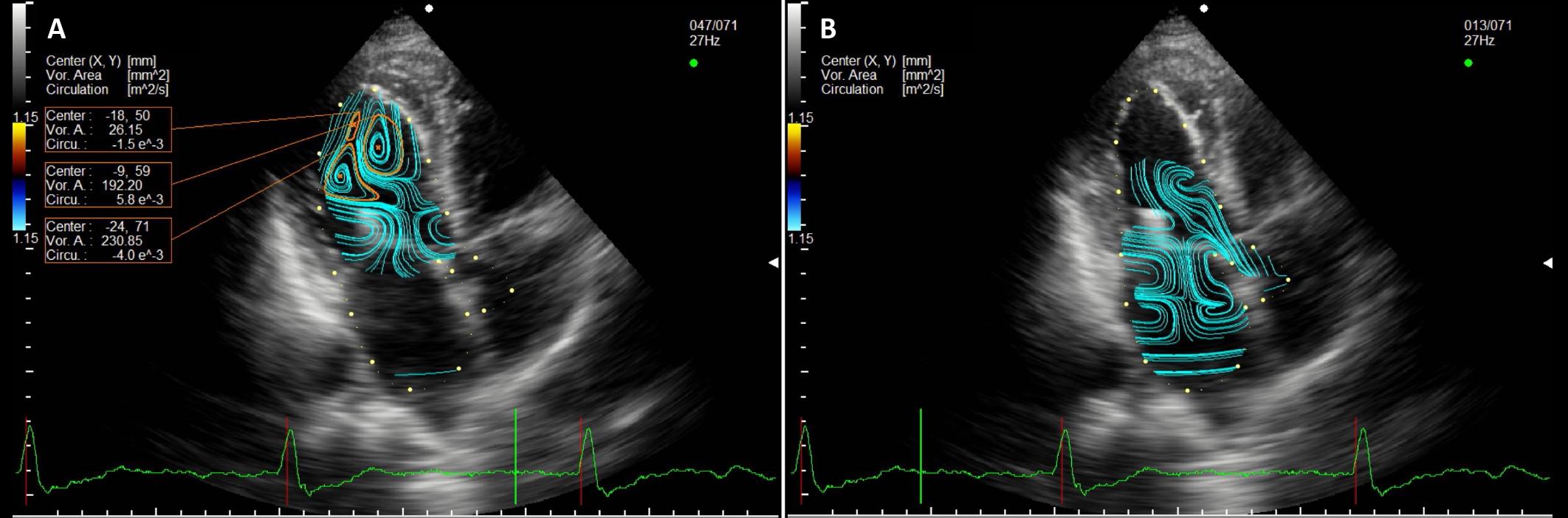

Supplement: Supplementary Figure S7 — Apical long-axis view. Intracardiac vortices in early diastole (A) and mid systole (B) after mitral valve replacement with mechanical prosthesis. [file Image7.jpeg]

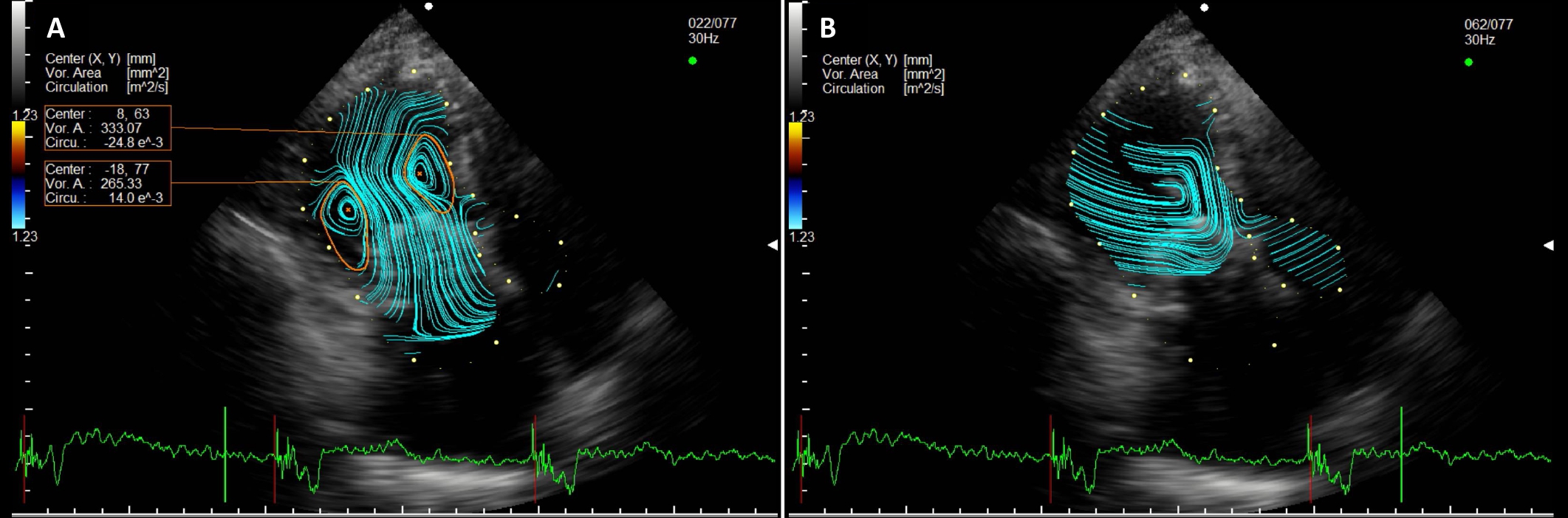

Supplement: Supplementary Figure S8 — Apical long-axis view. Intracardiac vortices in early diastole (A) and mid-systole (B) after transcatheter mitral valve replacement with Tendyne system. [file Image8.jpeg]

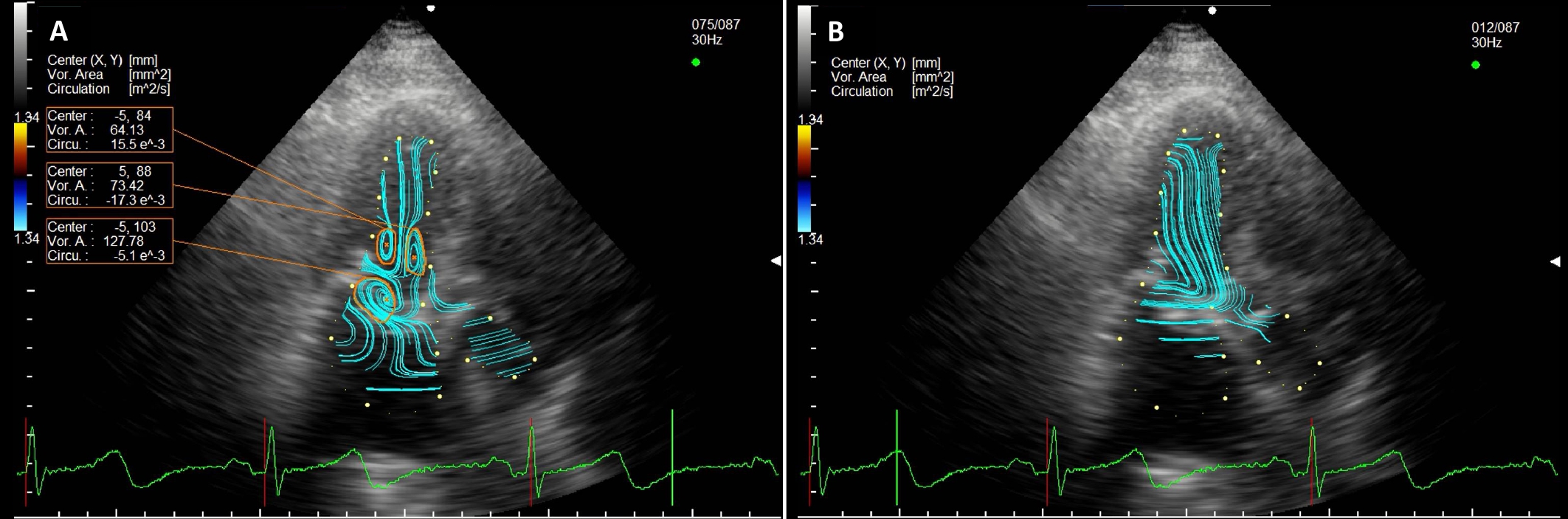

Supplement: Supplementary Figure S9 — Apical long-axis view. Intracardiac vortices in early diastole (A) and mid-systole (B) after surgical implantation of a Sapien 3 valve in mitral annular calcification (MAC). [file Image9.jpeg]

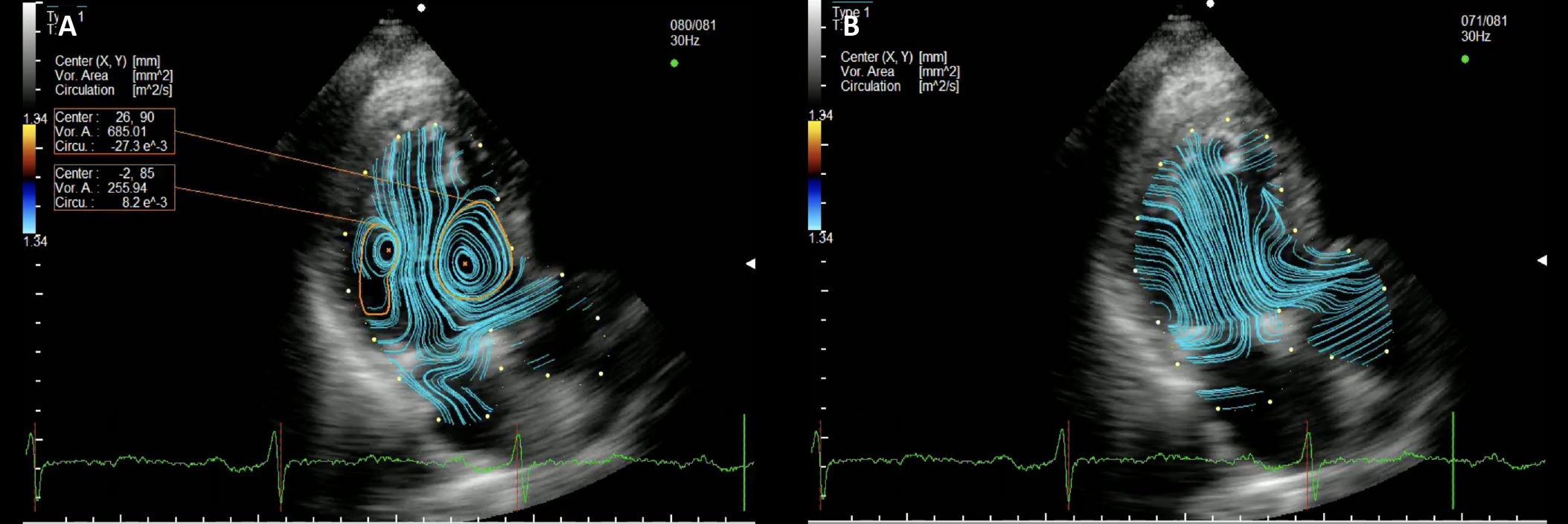

Supplement: Supplementary Figure S10 — Apical long-axis view. Intracardiac vortices in early diastole (A) and mid-systole (B) after transapical off-pump mitral valve repair with NeoChord TM device. [file Image10.jpeg]
