# Supplementary material for: Flow dynamic assessment of native mitral valve, mitral valve repair and mitral valve replacement using vector flow mapping intracardiac flow dynamic in mitral valve regurgitation
Source: Front Cardiovasc Med. 2023 Mar 24;10:1047244. doi: 10.3389/fcvm.2023.1047244 (PMC10080047; doi:10.3389/fcvm.2023.1047244)
Supplement: Supplementary file 31 [file Table1.docx]

**Table**. Ultrasound evaluation.

| **Variable** | **Normal MV** | **Degenerative MR** | **Functional MR** | **MV repair** | **TEER** | **MV replacement** | **MV replacement** | **TMVR** | **Sapien 3 in MAC** | **NeoChord TM Device** |
| --- | --- | --- | --- | --- | --- | --- | --- | --- | --- | --- |
| **Gender** | Female | Male | Female | Male | Male | Male | Female | Female | Female | Male |
| **Age, years** | 54 | 75 | 73 | 60 | 79 | 44 | 71 | 72 | 70 | 49 |
| **Technical procedure** | - | - | - | 1 pair of artificial chord for P2+Medtronic Simulus 36 annuloplasty ring | transcatheter mitral edge-to-edge with 2 MitraClips | Medtronic Mosaic 33 | Corcym Carbomedics 29 | Transcatheter Tendyne LP 33S | surgical Sapien 3 29 | Transapical off-pump implantation of 3 pairs of Neochords for P2 |
| **Conventional echocardiography** |  |  |  |  |  |  |  |  |  |  |
| LVMi, g/m^2^ | 75 | 110 | 122 | 100 | 159 | 136 | 90 | 120 | 113 | 150 |
| LVEDV, mL | 80 | 145 | 149 | 148 | 198 | 185 | 110 | 138 | 124 | 181 |
| LV Ejection fraction, % | 60 | 60 | 45 | 60 | 42 | 37 | 59 | 40 | 67 | 60 |
| Mitral E wave, cm/s | 80 | 110* | 150 | 87 | 105 | 110 | 135 | 100 | 125 | 84 |
| Average e', cm/s | 11 | 16 | 6 | 9 | 7 | 8 | 7 | 7 | 6 | 10 |
| Average E/e’ | 7 | 7 | 25 | 10 | 15 | 14 | 19 | 14 | 21 | 11 |
| LAVi, mL/m^2^ | 19 | 38 | 49 | 42 | 52 | 41 | 40 | 42 | 47 | 32 |
| TAPSE, mm | 21 | 20 | 18 | 16 | 19 | 15 | 17 | 17 | 17 | 17 |
| Systolic PAP, mmHg | 20 | 35 | 59 | 35 | 38 | 40 | 35 | 38 | 40 | 22 |
| **Vector Flow Mapping** |  |  |  |  |  |  |  |  |  |  |
| Energy loss, J/m∙s* | 0.36 | 0.51 | 0.65 | 0.39 | 0.67 | 0.48 | 0.55 | 0.47 | 0.74 | 0.41 |

*expressed as the mean energy loss of three complete cardiac cycles.

EDV: end-diastolic volume; LAVi: left atrial volume index; LV: left ventricle; LVMi: left ventricle mass index; PAP: pulmonary artery pressure; TAPSE: tricuspid annular plane systolic excursion, MV: mitral valve; MR: mitral regurgitation; TEER: transcatheter edge-to-edge repair; TMVR: transcatheter mitral valve replacement; MAC: mitral annulus calcification
